# Detection of Tick-Borne Pathogens in Lambs Undergoing Prophylactic Treatment Against Ticks on Two Swedish Farms

**DOI:** 10.3389/fvets.2018.00072

**Published:** 2018-04-16

**Authors:** Giulio Grandi, Anna Aspán, Jenny Pihl, Katarina Gustafsson, Fredrik Engström, Tomas Jinnerot, Robert Söderlund, Jan Chirico

**Affiliations:** ^1^Department of Microbiology, National Veterinary Institute (SVA), Uppsala, Sweden; ^2^Department of Biomedical Sciences and Veterinary Public Health, Section for Parasitology, Swedish University of Agricultural Sciences (SLU), Uppsala, Sweden; ^3^Farm and Animal Health, Uppsala, Sweden

**Keywords:** tick prophylaxis, tick-borne pathogens, sheep, serology, polymerase chain reaction, 16S rRNA sequencing

## Abstract

Tick-borne pathogens (TBPs), especially *Anaplasma phagocytophilum*, cause disease in grazing livestock. Tick prophylaxis is, therefore, a routine practice in sheep flocks in Sweden, especially in central, southern, and coastal areas of the country where ixodid ticks (*Ixodes ricinus* and *Haemaphysalis punctata*) are present. In the present study, the status of infection by *A. phagocytophilum* and other TBPs in lambs treated with tick prophylaxis has been assessed serologically and with polymerase chain reaction (PCR). Blood samples (*n* = 78) from lambs (*n* = 20) subjected to regular tick prophylactic treatment (flumethrin, Bayticol^®^) at two sites in different regions in Sweden (Östergötland, Gotland) were collected on four occasions from May until July 2013. The severity of clinical signs in *Anaplasma-*infected animals is known to differ between these two regions. In total, 20% of blood samples were PCR-positive for *A. phagocytophilum*. Serological analyses showed that 33% of all collected samples were positive for *A. phagocytophilum*, while 2.5% were positive for *Borrelia burgdorferi* s.l. and 13% for tick-borne encephalitis virus (TBEV). Percentages of lambs positive were 75 and 45% for *A. phagocytophilum* antibodies and DNA, respectively, while 10 and 45% were serologically positive for *B. burgdorferi* s.l. and TBEV, respectively. Sequencing of partial 16S rRNA genes from *Anaplasma* PCR positive samples revealed presence of *A. phagocytophilum* in all animals in Östergötland, while sequences consistent with *A. phagocytophilum* as well as *A. capra* and *A. bovis* were found on the island of Gotland. This is the first report of the occurrence of the latter two species in Sweden.

## Introduction

Ixodid ticks are spreading in many geographical areas, including northern Europe. The reasons for this complex phenomenon are not fully understood (1). The consequences of a more abundant population of ticks in terms of increased possibilities for tick-borne pathogens (TBPs) to circulate within and between geographical regions are also currently unclear. The incidence of human cases of certain tick-borne diseases has been rising during the last decades; an example of this is encephalitis caused by tick-borne encephalitis virus (TBEV) (2). Furthermore, while it has been observed an increase of the incidence of Lyme borreliosis in humans in southern Sweden associated with warmer winters (3), to the authors’ knowledge, there is no similar information available for anaplasmosis. Also, sheep are regularly exposed to ticks in Europe and in Sweden, where the most abundant tick (*Ixodes ricinus*) can transmit different TBPs including *Anaplasma phagocytophilum*. Sheep can also be sentinels for *Borrelia burgdorferi* sensu lato. Recently, a clinical case in a sheep naturally infected by TBEV was described (4). In addition to the zoonotic potential of all these pathogens, *A. phagocytophilum* is a major threat to the sheep industry since it can cause tick-borne fever (TBF). Another important feature of *A. phagocytophilum* infection is the associated immunosuppression that can lead to further infections and complications (5). Tick prophylaxis of sheep is regularly applied in the Nordic countries (6). The aim of the present study is to describe the presence of infection of the most common TBPs in Swedish lambs treated with tick prophylaxis and kept on pastures in two different locations, characterized by different dominant tick species [*I. ricinus* and *Haemaphysalis punctata* (7)]. The same analyses were performed on lambs showing poor growth in order to investigate the role of TBPs in animals protected by tick prophylaxis but showing clinical signs. Furthermore, partial sequencing of the 16S rRNA genes of the bacterial strains found in sheep in these two locations was performed in order to understand the ecology of *A. phagocytophilum*. Contacts between wild and domesticated animals in the Nordic region are extremely frequent as most pastures in Sweden are surrounded by natural forests and farm lands outside urban areas. As the role of wildlife as a reservoir of TBPs for domesticated animals and humans in Europe is not fully understood, it is important to assess the effects of tick prophylaxis in such a complex ecological context.

## Materials and Methods

### Study Area, Farms and Tick Prophylaxis Protocol

The study was performed between May and July, 2013. Farms included in the study were located in two different areas, one near Linköping in the county of Östergötland “L” (58°15′39.2′′N 15°39′59.2′′E) and one on the island of Gotland “G” (57°54′24.1′′N 19°6′27.0′′E). In both of these areas, tick prophylaxis is recommended by the consulting veterinarians at Farm and Animal Health, an advisory company with the aim of preventing animal health problems and improving animal welfare. Inclusion criteria for the farms were documented problems of anaplasmosis and flock size (≥100 grazing lambs). Farm L, which had been regularly applying tick prophylaxis for at least 2 years before the study, was selected because high lamb mortality and morbidity was recorded in 2011. At that time, the majority of lambs were treated with antibiotics and DNA of *A. phagocytophilum* was demonstrated by Polymerase Chain Reaction (PCR) in blood samples; also, pathological signs of anaplasmosis were found at necropsy in one lamb. In 2012, at least 13 lambs died and at necropsy of four lambs, three had splenomegaly and one was positive at *A. phagocytophilum* PCR. Farm “G” was selected because of findings of anaplasmosis at necropsy in 2012, associated with sudden death of lambs on pasture. Responsible veterinarians had recorded tick-related problems at site G since 2005. The flocks of lambs at both sites were treated with flumethrin pour on (Bayticol^®^, Bayer Animal Health, Leverkusen, Germany) at turn out and then every 3 weeks, until the end of June/beginning of July 2013. The dose rate was 2 mg/kg bodyweight. The time-points of tick prophylaxis were the following: T1 (05/05/2013—site L, 15/05/2013—site G), T2 (29/05/2013—site L, 05/06/2013—site G), and T3 (18/06/2013—site L, 01/07/2013—site G).

### Animals

Ten lambs born in the spring of 2013 (at their first grazing season) were randomly selected on each site (total, *n* = 20) and were then followed up for a period of 9–10 weeks (see Sample Collection). Moreover, lambs (*n* = 15 on site L and *n* = 8 on site G) that were showing poor growth (<150 g/day) between the visits to the farms were blood sampled once. These extra lambs were sampled on site L at T2 (*n* = 5), T3 (*n* = 5), and T4 (*n* = 5) and on site G at T3 (*n* = 5) and T4 (*n* = 3).

### Sample Collection

Blood samples were collected from the jugular vein. A total of 101 blood samples were collected. One batch of blood samples (*n* = 78) was collected from the 10 lambs recruited on each site at the same three time points used for the application of tick prophylaxis, plus at an additional time-point, T4 (08/07/2013—site L, 17/07/2013—site G), around 2 weeks after the last prophylactic treatment. Totally, four blood samples were collected from each lamb, with the exception of one sample from one lamb at site L (T2) and one sample from one lamb at site G (T3) that could not be collected. Blood was collected in EDTA tubes and serology tubes; the latter were centrifuged to get serum, and all the material (EDTA blood + serum) was stored at −20°C until analyzed.

### Serological Analyses

Sera were analyzed using an indirect immunofluorescence antibody assay (IFA) to determine the antibody titer to an equine variant of *A. phagocytophilum* (formerly *Ehrlichia equi*) (8, 9). Briefly, twofold dilutions of sera were added to slides pre-coated with *A. phagocytophilum* antigen (Protatec, St. Paul, MN, USA). Bound antibodies were visualized by fluorescein-isothiocyanate-conjugated rabbit-anti-sheep immunoglobulin (Cappel, Organon Teknika, West Chester, PA, USA). Sera were screened for antibodies at dilution 1:40.

Antibodies to *B. burgdorferi* s.l. were also tested with an IFA, following the same protocol using an in-house test. The slides were pre-coated with a Swedish strain, *B. burgdorferi afzelii* ACA 1, a species within the *B. burgdorferi* s.l. group (10). This strain has been found to show cross-reactivity with *B. garinii* and *B. burgdorferi* sensu stricto according to the annual quality assurance program of the National Veterinary Institute (SVA, Uppsala), in cooperation with other European laboratories. The cut-off value for the *Borrelia* IFA was a 1:80 dilution.

Serology against TBEV was performed using an enzyme-linked immunosorbent assay implemented in the kit Immunozym FSME IgG all species (PROGEN Biotechnik GmbH, Heidelberg, Germany). The kit instructions were used without modifications and the plates were read with a Multiskan EX (Thermo Fisher Scientific, Stockholm, Sweden) using the 450 nm filter.

### PCR for *A. Phagocytophilum*

DNA was extracted from 200 ml EDTA blood samples using the EZ1 DNA tissue kit in the Biorobot EZ1 with the EZ1 DNA bacterial card, according to the manufacturer’s instruction (QiaGen, Hilden, Germany). To prevent contamination when performing PCR analysis, preparation of reaction mixtures, DNA extraction, and amplification were performed in different laboratory rooms. Additionally, aerosol-resistant filter pipette tips were used throughout all experiments. Primers used for real-time PCR targeting the 16s rRNA gene of *A. phagocytophilum* were the ones described by Goodman et al. (11) and in the erratum of the paper, with the addition of a TaqMan probe [6-FAM(CTGTCGTCAGCTCGTGTCGTGAGATGTTG)BHQ-1]. Each 15-µl PCR reaction mixture contained 1× PerfeCTa qPCR FastMix, UNG, Low Rox (Quanta BioSciences Inc., Gaithersburg, MD, USA), 0.5 µM of each primer, 0.1 µM of the TaqMan-probe, and 2 µl of template DNA. The PCR amplification was performed in an Applied Biosystems 7500 Fast Real-Time PCR System (Applied Biosystems, Carlsbad, CA, USA), using the cycling parameters: 95°C for 3 min, then 45 cycles of 95°C for 3 s, and 60°C for 30 s.

### Species Confirmation

From all real-time PCR positive blood samples, Sanger sequencing of the *Anaplasma* 16S rRNA gene was done as previously described (12). Deep sequencing of partial *Anaplasma* 16S rRNA sequences was performed using the Illumina 16 S protocol for the MiSeq system (https://www.illumina.com/…/illumina…/16s/16s-metagenomic-library-prep-guide-15044223-b.pdf, accessed 11/12/2017) with custom primers MiSeq_16SAna_F 5′-TCGTCGGCAGCGTCAGATGTGTATAAGAGACAGGAAAGCGTGGGGAGCAAACA-3′ and MiSeq_16SAna_R 5′-GTCTCGTGGGCTCGGAGATGTGTATAAGAGACAGGTAGCACGTGTGTAGCCC-3′. Reads pairs were merged using COPE (13), primer sequences removed, and the number of high-quality observations counted for each sequence variant in each sample. Any variant with a count <5% of the most common variant in the same sample was filtered out to remove sequence error artifacts. A negative control without any clinical sample material was prepared, sequenced, and analyzed to detect low-level bacterial DNA contamination commonly found in reagents (14). All observations of *Anaplasma* variants were supported by >1,800 sequencing read pairs in each sample.

## Results

### Serology

Serology results are summarized in Table 1. One lamb from each farm had antibodies against *B. burgdorferi* s.l. at one time-point. Regarding TBEV, six lambs at site G and three lambs at site L were borderline positive/positive at least once, with one lamb at site G being positive at two time-points. Antibodies against *A. phagocytophilum* were found in six and nine lambs at sites G and L, respectively. At site G, most positive lambs were positive only at the first sampling (T1). Also at site L, the majority of lambs were positive at T1, but a pattern of increasing exposure to *A. phagocytophilum* was observed toward the end of study period.

**Table 1:** Results of serological analyses.

Animal ID	T1			T2			T3			T4		
	Ap	Bb	Tick-borne encephalitis virus (TBEV)	Ap	Bb	TBEV	Ap	Bb	TBEV	Ap	Bb	TBEV
G1	+	–	–	–	–	–	–	–	–	–	–	–
G2	–	–	–	–	–	–	–	–	–	–	–	–
G3	+	–	(+)	+	–	–	–	–	–	–	–	–
G4	–	–	–	–	–	–	–	–	–	–	–	–
G5	+	–	–	–	–	(+)	–	–	–	–	–	–
G6	–	–	(+)	–	–	–	–	–	–	–	–	–
G7	–	–	+	–	–	(+)	–	–	–	–	–	–
G8	–	–	(+)	–	–	–	+	–	–	–	+	–
G9	+	–	(+)	–	–	–	NA	NA	NA	–	–	–
G10	+	–	–	–	–	–	–	–	–	–	–	–
*n* = positive (site G)	5	0	5	1	0	2	1	0	0	0	1	0
L1	+	–	–	–	–	–	–	–	–	–	–	–
L2	+	+	–	–	–	–	–	–	–	–	–	–
L3	–	–	–	–	–	–	+	–	–	+	–	(+)
L4	+	–	–	–	–	–	–	–	–	–	–	–
L5	+	–	–	–	–	–	+	–	–	+	–	–
L6	+	–	–	–	–	–	–	–	–	+	–	–
L7	+	–	–	+	–	–	+	–	–	+	–	–
L8	+	–	–	NA	NA	NA	+	–	–	+	–	(+)
L9	+	–	–	–	–	–	–	–	–	–	–	–
L10	+	–	–	–	–	–	–		–	–	–	+
*n* = positive (site L)	9	1	0	1	0	0	4	0	0	5	0	3
*n* = positive (total G + L)	14	1	5	2	0	2	5	0	0	5	1	3

*(+) indicates TBEV borderline positive samples. Ap, *Anaplasma phagocytophilum*; Bb, *Borrelia burgdorferi* s.l.; TBEV, tick-borne encephalitis; T1–T4, sampling occasions (see Materials and Methods); G, lambs from the island of Gotland; L, lambs from the mainland*.

The following co-infection patterns as demonstrated by positive serology were found: *B. burgdorferi/A. phagocytophilum* (lamb L2 at T1), *A. phagocytophilum*/TBEV (*n* = 4 lambs: G3 and G9 at T1, L3 and L8 at T4). Regarding serology in lambs that showed poor growth during the visits T2–T3–T4, 10 lambs on site L were positive for *A. phagocytophilum* and two were positive for both TBEV and *A. phagocytophilum*, while none from site G had antibodies against the considered TBPs.

### DNA Detection and 16S rRNA Sequencing

Results of PCR analyses are summarized beside serological results for the same pathogen in Table 2. Blood samples of four lambs at site G and five lambs at site L were PCR-positive for *A. phagocytophilum*, with two lambs (G6 and G10) at site G which were positive at three time-points and three lambs (L5, L6, L7) at site L positive on two occasions. Among lambs showing poor growth, seven were positive for *A. phagocytophilum* at site L, while two were positive at site G.

**Table 2:** Results of serology (Ab) and polymerase chain reaction (PCR) analyses for *Anaplasma phagocytophilum*.

Animal ID	T1		T2		T3		T4	
	PCR	Ab	PCR	Ab	PCR	Ab	PCR	Ab
G1	–	+	–	–	–	–	+	–
G2	–	–	–	–	–	–	–	–
G3	–	+	–	+	–	–	–	–
G4	–	–	–	–	–	–	–	–
G5	–	+	–	–	–	–	+§	–
G6	–	–	+	–	+	–	+§	–
G7	–	–	–	–	–	–	–	–
G8	–	–	–	–	–	+	–	–
G9	–	+	–	–	NA	NA	–	–
G10	+§	+	+	–	+	–	+§	–
*n* = positive (site G)	1	5	2	1	2	1	3	0
L1	–	+	–	–	–	–	–	–
L2	–	+	–	–	–	–	–	–
L3	–	–	+§	–	–	+	–	+
L4	–	+	–	–	–	–	–	–
L5	–	+	+§	–	–	+	(+)	+
L6	–	+	–	–	+§	–	+	+
L7	NA	+	+§	+	+	+	–	+
L8	–	+	NA	NA	–	+	–	+
L9	–	+	–	–	–	–	–	–
L10	–	+	+§	–	–	–	–	–
*n* = positive (site L)	0	9	4	1	2	4	2	5
*n* = positive (total G + L)	1	14	6	2	4	5	5	5

*§, subjected to 16s rRNA amplicon sequencing; +, indicates weak positive PCR reaction. T1–T4, sampling occasions (see Materials and Methods); G, lambs from the island of Gotland; L, lambs from the mainland*.

Sanger sequencing of real-time PCR positive blood samples from all animals at site L were 100% identical with the 16S rRNA sequences of *A. phagocytophilum* strain Norway variant 2 (CP015376.1). However, samples collected from site G gave dubious Sanger sequencing results. Therefore, amplicon sequencing of part of the 16s rRNA gene was performed on selected samples (Table 3). From samples from site L (*n* = 4), all sequences (437 bp) were 100% identical to *A. phagocytophilum* strain Norway variant 2 (CP015376.1). In contrast, the sequences from site G (*n* = 6) exhibited three variants; 1. identical to *A phagocytophilum* strain ES34 (AB196720), 2. identical to *A. capra* isolate S63a (MF066918), and 3. identical to *A. bovis* strain WHHLHP-119 (ID: KX987337). The negative sequencing control revealed the presence of low levels of reagent contamination by *Pseudomonas* sp., *Serratia*/*Enterobacter*/*Leclercia* sp., and *Lactococcus* sp. Some of these bacteria were also detected in a few of the clinical samples, but at low levels unlikely to affect *Anaplasma* detection.

**Table 3:** Results of matching of sequences from samples on site G (Gotland), including two blood samples from lambs showing poor growth (EG1, EG2).

Animal ID	Timepoint	Amplicon sequencing results similar to:		
G5	T4			*Anaplasma phagocytophilum*, strain ES34
G6	T3			*A. phagocytophilum*, strain ES34
G10	T1	*A. capra*, isolate S63a	*A. bovis*, strain WHHLHP-119	
G10	T4	*A. capra*, isolate S63a		
EG1	T3	*A. capra*, isolate S63a		
EG2	T4		*A. bovis*, strain WHHLHP-119	

## Discussion

All pathogens studied were directly or indirectly detected, despite the fact that examined sheep populations were treated with flumethrin. Active *A. phagocytophilum* infection among sheep protected against ticks by flumethrin had been described before (6), but no clear explanation has been found for this partial lack of efficacy of the treatment.

The high number of lambs showing antibodies against *A. phagocytophilum* and TBEV during the first sampling time point is probably due to passive transfer of maternal antibodies and is in agreement with findings of Stuen et al. (6). *Anaplasma* DNA was found in a single animal at T1, and the proportion of positive lambs increased at the later sampling time points.

Active infection of TBPs is demonstrated by the detection of antibodies later during the study; for example, five and three lambs at site L were positive, respectively, for *A. phagocytophilum* and TBEV in July 2013. Another demonstration of the presence of active infection is the detection of *A. phagocytophilum* DNA, in some cases preceding the appearance of specific antibodies (lambs L3 and L5).

While there was a certain consistency between serological and PCR results for *A. phagocytophilum* at site L (all but one of the lambs positive by PCR had specific antibodies against the bacteria at T2–T3–T4), this is not true for certain lambs at site G (G6 and G10), which despite observations of persistent infection on several occasions never seroconverted. However, as further discussed below animals on Gotland revealed to be infected by other and multiple species of *Anaplasma*, possibly reducing the sensitivity of the serological assay.

The small number of samples serologically positive for *B. burgdorferi* s.l. does not allow estimation of the level of exposure in the considered population. Regarding TBEV, only two lambs in the present study were positive, and at different time-points. However, if borderline TBEV samples were included as many as nine lambs had antibodies both at site G and L. The interpretation of borderline samples is somehow problematic, but previous studies have confirmed that they may be true but low positive by serum neutralization (15).

In the summer of 2013 when the two farms were sampled, there were few clinical cases of TBF among sheep in Sweden (Olof Schwan, personal communication). However, in general, sheep farmers on Gotland report more severe disease in lambs, than farmers on the mainland. The reason for this is unknown. On mainland Sweden, the dominant tick species is *I. ricinus*, while on the island of Gotland, *H. punctata* is also commonly found (7). Whether the pathogens harbored by these two tick species differ has not yet been studied. Still, results from the sequencing indicated the presence of multiple *Anaplasma* species of in lambs on Gotland (i.e., *A. phagocytophilum, A. capra*, and *A. bovis*), while only *A. phagocytophilum* was identified in the mainland flock. While species/strain confirmation was based on a short region of the 16S rRNA genes and the species assignments should be considered preliminary, our results strongly indicate that lambs on Gotland are exposed to a wider range of *Anaplasma* variants, but also seems to be at a higher risk of being co-infected by multiple species. This may explain the difference in disease manifestations between the mainland and the island of Gotland. The ability to detect multiple genotypes of a pathogen species present at different quantities in a sample has been significantly improved in recent years with the adaptation of the type of high-throughput sequencing technology, which was used in the present study. Implementation of these techniques on more infected animals as well as on ticks is warranted to better understand the ecology of different *Anaplasma* genotypes and their role in causing disease in the studied regions.

The different kind of wildlife present in the two investigated areas could also be a factor involved in the difference of circulation of TBPs in the studied populations. On site L, roe deer are abundant, acting as an important blood source for ticks, while this species has only been recently introduced to Gotland, and is less abundant there. While the patterns of circulation of different strains of *A. phagocytophilum* between wildlife and domestic animals have been extensively investigated in the USA, there are still many gaps regarding our understanding of the situation in Europe (5).

The role of roe deer as a source of *A. phagocytophilum* infection for humans and/or domesticated animals is still under debate. Some studies hypothesize that roe deer may harbor bacterial strains, which are different from those in sheep (16), while others hypothesize that roe deer could contribute to the spread of the bacteria or act as reservoir of *A. phagocytophilum* for ruminants in general (17).

Another important difference between the studied regions is the presence of wild rabbits (*Oryctolagus cuniculus*) on the island of Gotland, while they are absent from the area of Linköping. Studying the role of wild rabbits as the potential reservoir for the *Anaplasma* strains, which were unique at Gotland, and for other TBPs, could contribute to a more complete picture of the ecology of TBPs in this and other areas. To the authors’ knowledge, this is the first time that infections by *A. bovis* and *A. capra* are recorded in Sweden. It has already been demonstrated that, in isolated ecosystems, the circulation of *B. burgdorferi* s.l. relies on different hosts than on the mainland: in a study conducted on two Swedish islands, the maintenance host for the *Borrelia* spirochetae was the mountain hare (*Lepus timidus*) (18).

More studies are needed in order to better understand the peculiar situation of the island of Gotland, since both different clinical presentation in lambs, tick species, and available wild hosts are creating a unique ecosystem. The observation of different *Anaplasma* species in this geographical area is complicating the puzzle and is also confirming that further investigations are needed.

## Ethics Statements

This study was performed in the frame of the veterinary services provided by “Farm and Animal Health” company, so all the procedures, including tick prophylaxis and blood samplings were performed by company veterinarians during their visits to the farms and with the purposes of checking the health status within these farms. Animal owners were customers of the “sheep health” program of “Farm and Animal Health” and both the visits and the procedures were included in the agreement between the owners and the company.

## Author Contributions

JC, KG, and AA designed the study. KG and FE performed field sampling. AA, TJ, RS, and JP performed molecular analyses. AA, TJ, and RS analyzed sequences. GG performed serological analyses. GG and AA interpreted the results and wrote the manuscript. All authors read and approved the final manuscript.

## Conflict of Interest Statement

The authors declare that the research was conducted in the absence of any commercial or financial relationships that could be construed as a potential conflict of interest.
